# The Use of Virtual Reality Interventions to Promote Positive Mental Health: Systematic Literature Review

**DOI:** 10.2196/44998

**Published:** 2023-07-06

**Authors:** Giorgio Li Pira, Beatrice Aquilini, Alessandro Davoli, Silvana Grandi, Chiara Ruini

**Affiliations:** 1 Department of Psychology University of Bologna Bologna Italy; 2 Department for Life Quality Studies University of Bologna Rimini Italy

**Keywords:** positive mental health, well-being, virtual reality, interventions, psychopathology, mobile phone

## Abstract

**Background:**

A large body of research has documented the efficacy of psychological interventions integrated with virtual reality (VR) therapies in treating psychiatric disorders. However, the concept of positive mental health calls for a 2-fold approach in which both symptoms and positive functioning should be addressed by modern interventions.

**Objective:**

This review aimed to summarize studies that applied VR therapies by embracing the positive mental health perspective.

**Methods:**

A literature search was conducted by entering the following keywords—“virtual reality” AND “intervention” OR “treatment” OR “therapy” AND “mental health” NOT “systematic review or meta-analysis”—and limiting it to “journal article” and the English language. To be included in this review, articles had to present at least one quantitative measure of positive functioning and one quantitative measure of symptoms or distress and had to investigate adult populations, including populations with psychiatric disorders.

**Results:**

A total of 20 articles were included. They described various VR protocols that were applied for the treatment of anxiety disorders (5/20, 25%), depression (2/20, 10%), posttraumatic stress disorder (3/20, 15%), psychosis (3/20, 15%), and stress (7/20, 35%). Most of the studies (13/20, 65%) showed the beneficial effects of VR therapies in improving stress and negative symptoms. However, 35% (7/20) of the studies showed no or a small effect on the various dimensions of positivity, particularly in clinical samples.

**Conclusions:**

VR interventions might be cost-effective and largely scalable, but further research is needed to develop existing VR software and treatments according to the modern positive mental health approach.

## Introduction

### Background

The implementation of virtual reality (VR) programs in psychotherapy has been increasing over the past 3 decades [1-3], especially after the past COVID-19 pandemic (Table 1). Since the early 1990s, VR has been used to treat disorders such as specific phobias, panic disorder, and posttraumatic stress disorder (PTSD). Although the technology was quite raw, it was sufficiently capable of producing convincing 3D images inside a head-mounted display (HMD) or inside a room where the images were projected on the walls (cave automatic virtual environment) [4]. Today, many protocols are available for the treatment of different psychopathologies, from VR exposure therapy (VRET) to protocols dedicated to eating disorders, depression, obsessive-compulsive disorder (OCD), and psychosis.

**Table 1:** Cumulative record of publications in the PsycInfo database under the keywords phrase “VR intervention” from the years 2012 to 2022.

Year	Cumulative record of publications, n
2012	7
2013	9
2014	11
2015	6
2016	4
2017	10
2018	17
2019	28
2020	52
2021	63
2022	31

However, the current definition of mental health, which emerged from the positive psychology perspective, entails 2 related components: symptoms or psychopathology and well-being and positive functioning [5,6]. According to this model, psychopathology and well-being coexist along a continuum where the states of mental illness and languishing are the negative components in contrast to the state of flourishing, which is the positive component. Languishing is defined as a state of emptiness in which the individual experiences few instances of well-being and is considered vulnerable to the development of psychiatric disorders. Flourishing, in contrast, is also labeled as optimal human functioning and is characterized by the presence of high levels of emotional, psychological, and social well-being. The treatment of symptoms and distress has been the main focus of the mental health agenda over the past decades. However, according to this complete mental health model, the promotion of well-being and optimal human functioning should receive the same attention. According to the positive psychology perspective, positive psychological interventions can be conceived as “treatment methods” or intentional activities that aim to cultivate positive feelings, behaviors, or cognitions [7]. Longitudinal investigations have shown that cross-time gains in well-being predicted cross-time declines in mental illnesses and vice versa [8]. Various authors have observed that positive and negative characteristics can be considered at opposite ends of a single dimension and that their valence can vary according to the specific context [6,9,10]. For example, anger can become adaptive when it helps individuals reach their goals or fight for their ideas, depression can exist in the continuum with happiness, and it may become adaptive in encouraging people to change suboptimal aspects of their lives. Similarly, anxiety and calmness exist on the same continuum and are often assessed using similar items but with opposite coding procedures. As a consequence, mental health involves a complex balance of positive and negative psychological characteristics, and modern interventions should be able to address both components. Wood and Tarrier [9] proposed a positive clinical psychology framework in which clinical interventions may help individuals move away from symptoms and implicitly have an equal and opposite effect on well-being. Thus, the authors call for an integration between positive and clinical psychology in which psychological interventions should treat distress as well as they should promote well-being. According to Keyes [5], the promotion of well-being in the long run can be considered the most cost-effective mental health policy as it allows for the maintenance of optimal functioning in the population and prevents the onset of psychiatric disorders and the economic burden of expensive treatments.

Digital technologies can be considered valid tools to pursue this goal. Recently, with the advent of the pandemic, digital technologies have been used to deliver web-based psychotherapeutic interventions for addressing the psychological effects of COVID-19 or promoting positive functioning and well-being [11,12]. The latter applications can be subsumed under the umbrella of “Positive Technology” research [13].

As stated by Riva [13], “Positive Technology consists in the scientific and applied approach to the use of technology for improving the quality of our personal experience.” The core theoretical framework of this approach comes from the field of positive psychology and aims to use technology to manipulate the quality of experience, increase well-being, and generate strengths and resilience at the level of individuals, organizations, and society. Therefore, owing to “Positive Technology,” it is possible to study and understand how digital technologies can be used to promote health and well-being [14]. VR, owing to its ability to create controlled and tailored experiences that enhance the user’s sense of presence [13,15], has proven to be an important tool for increasing well-being. In particular, VR can affect 3 characteristics of personal experience that can promote personal well-being. VR can induce positive and pleasurable experiences; foster engagement and self-realization; and support and enhance connection and interpersonal relationships among individuals, groups, and organizations [16]. Studies conducted thus far have demonstrated the effectiveness of VR in several areas of application [17]. However, a limited body of research has used the complete mental health approach and tested whether modern, digitalized interventions were able to address symptoms and distress and promote well-being. A notable exception is a review published recently in this journal [18] that focused on the promotion of well-being through digital technologies. It found that self-help interventions improved well-being in young people aged 9 to 25 years. However, the review targeted the young population only. This population has very specific psychological features that change according to the stage of development of the participants. The results of this review cannot be generalized to the rest of the population. Similarly, other research groups have developed digital interventions for older adults and documented encouraging results for the promotion of positive mental health in the aging population [19-21].

### Objectives

This systematic review aimed to fill this literature gap by summarizing the most recent (last 10 years) research in the field. To the authors’ knowledge, the current literature lacks a systematic review of studies that provide a global assessment of the benefits of VR in treating symptoms and promoting well-being in adult populations. Therefore, the primary goal of this work was to collect and summarize scientific literature on the application of VR therapies according to the positive mental health framework, which includes not only data regarding the efficacy of VR treatments in improving symptoms but also data on the impact of these technologically advanced interventions in promoting positive functioning. In doing so, we hope to provide the reader with a broader understanding of the impact of these emerging digital therapies on the global mental health of adult individuals.

Considering the large number of private and public investments in digital technologies for mental health, this review may provide important initial data on their worthiness for promoting complete mental health in the adult population through the use of VR.

## Methods

### Design

A systematic review was conducted to extract recently published scientific papers that dealt with measures of positive functioning and symptoms and distress in adult populations. We focused on the last 10 years (from 2012 onward) as during that period, the Positive Technology research agenda was scientifically recognized and, since then, many VR protocols have been developed. This review followed the PRISMA (Preferred Reporting Items for Systematic Reviews and Meta-Analyses) guidelines [22].

### Search Method

A systematic literature search was conducted on the following electronic databases: EBSCOhost (PsycArticles and PsycInfo), PubMed, and Scopus.

The literature search was conducted by entering the following keywords: “virtual reality” AND “intervention” OR “treatment” OR “therapy” AND “mental health” NOT “systematic review or meta-analysis.”

### Selection Criteria

#### Inclusion

To be included in this review, the studies should involve (1) measures of positive functioning, (2) measures of symptoms and psychological distress, (3) adult populations, and (4) a description of a psychological intervention delivered using the VR system.

#### Exclusion

Studies were excluded if they (1) did not provide an assessment of positive functioning; (2) did not provide an assessment of symptoms; (3) were not experimental studies (eg, systematic reviews, protocols, or book chapters); (4) included only older adult or child populations; and (5) included adult populations with eating disorders, gambling, or substance use disorders. The latter clinical conditions were excluded as the source of positive emotions and well-being could be directly related to the exposure in the virtual environment to the objects of their addiction and could be a manifestation of the disorder itself [6,23,24]. Thus, individuals diagnosed with these clinical conditions may have a more complex balance between symptoms and positive functioning [23,25,26] and may require complex treatments with medications or further treatment ingredients [6,24,27,28].

### Data Collection

The titles and abstracts of the articles assessed for potential inclusion were identified and independently inspected by 1 reviewer, who excluded duplicates and articles that did not meet the inclusion criteria. All potentially relevant articles were then fully assessed by other 2 reviewers, who decided on inclusion. Uncertainty was resolved by coming to a consensus. Data were extracted using a predesigned template (Multimedia Appendix 1 [29-48]) with the following specified headings: study, sample size, outcome measures, treatment conditions, follow-up, main findings, and limitations.

### Risk of Bias and Quality of the Articles

To assess the bias of the individual studies, the following tools were used to sample a selection of quantitative studies: the Cochrane risk-of-bias tool [49] for between-group studies and the Standard Quality Assessment Criteria for Evaluating Primary Research Papers from a Variety of Fields [50] for single-arm or within-subject studies. The risk of bias was calculated by one of the authors for all the included articles.

## Results

### Search Outcome

**Figure 1:**
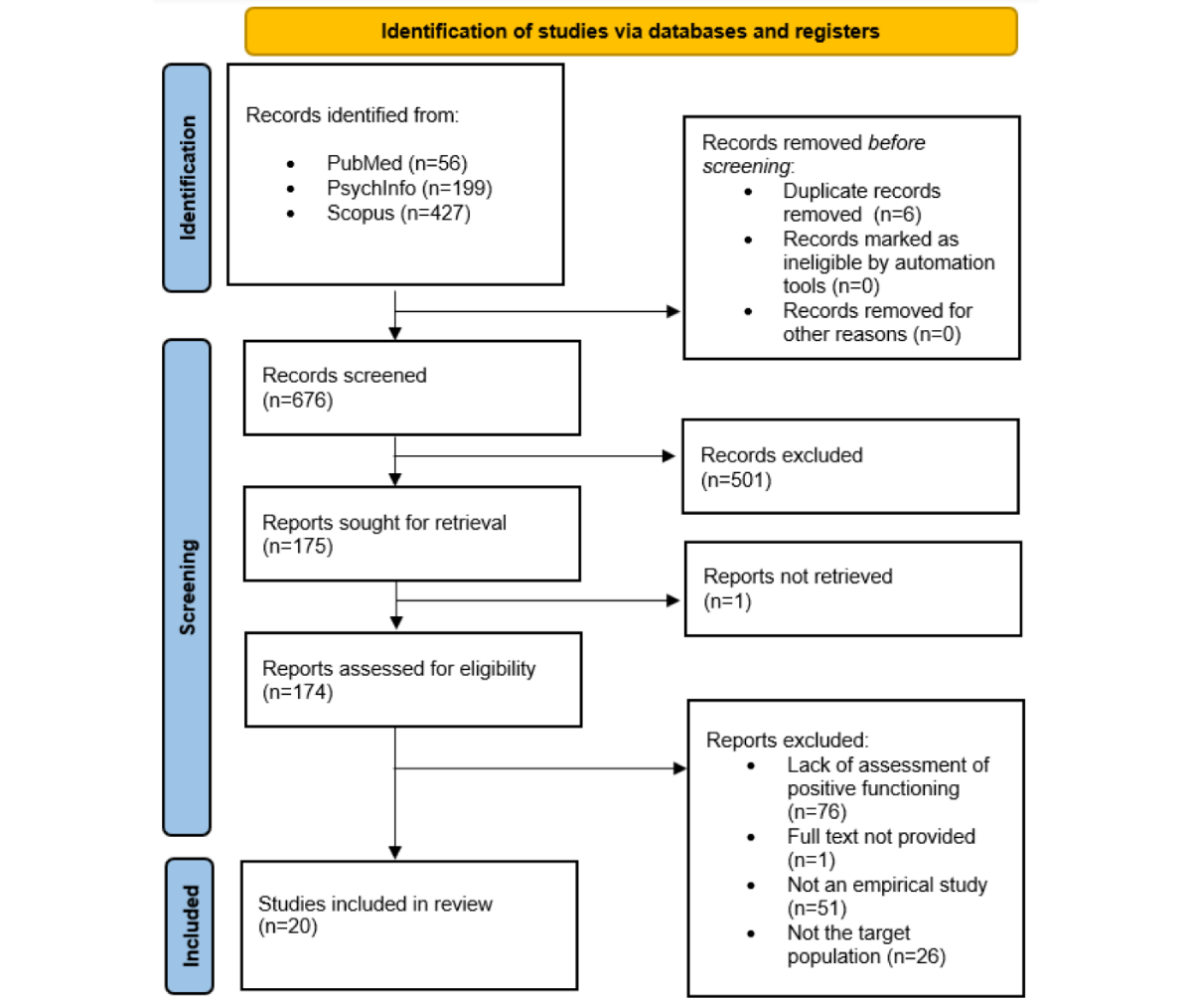
Flowchart depicting the identification and selection of the articles.

The first screening identified 682 articles. Most of them (626/682, 91.8%) were extracted from the EBSCOhost and Scopus databases. Only a few articles (56/682, 8.2%) were extracted from PubMed. After removing duplicates, 676 publications were identified and individually assessed based on the study title and the information provided in the abstract (see the review flowchart in Figure 1). Of these 676 papers, 501 (74.1%) were excluded according to the aforementioned inclusion and exclusion criteria, whereas the full texts of 174 (25.7%) articles were examined before the decision was made on whether to include them. Of these 174 studies, 76 (43.7%) did not assess well-being in combination with psychopathology or symptoms, 51 (29.3%) were not empirical studies, 26 (14.9%) did not include our target adult population, and it was not possible to obtain the full text of 1 (0.6%) paper; therefore, they were excluded. A total of 20 papers met all the inclusion criteria and were included in this review.

Multimedia Appendix 1 presents a summary of the findings from the 20 studies included in the review. Of these 20 studies, only 8 (40%) were randomized controlled studies, of which 3 (38%) used a waiting list as a control condition; 6 (30%) were pilot studies; 4 (20%) were case series or single-arm conditions; 1 (5%) had a within-subject design; and 1 (5%) had a between-subject design with 3 intervention groups. Hence, the quality of the included papers was low considering the high percentage of pilot and non–randomized controlled trial (RCT) studies.

**Figure 2:**
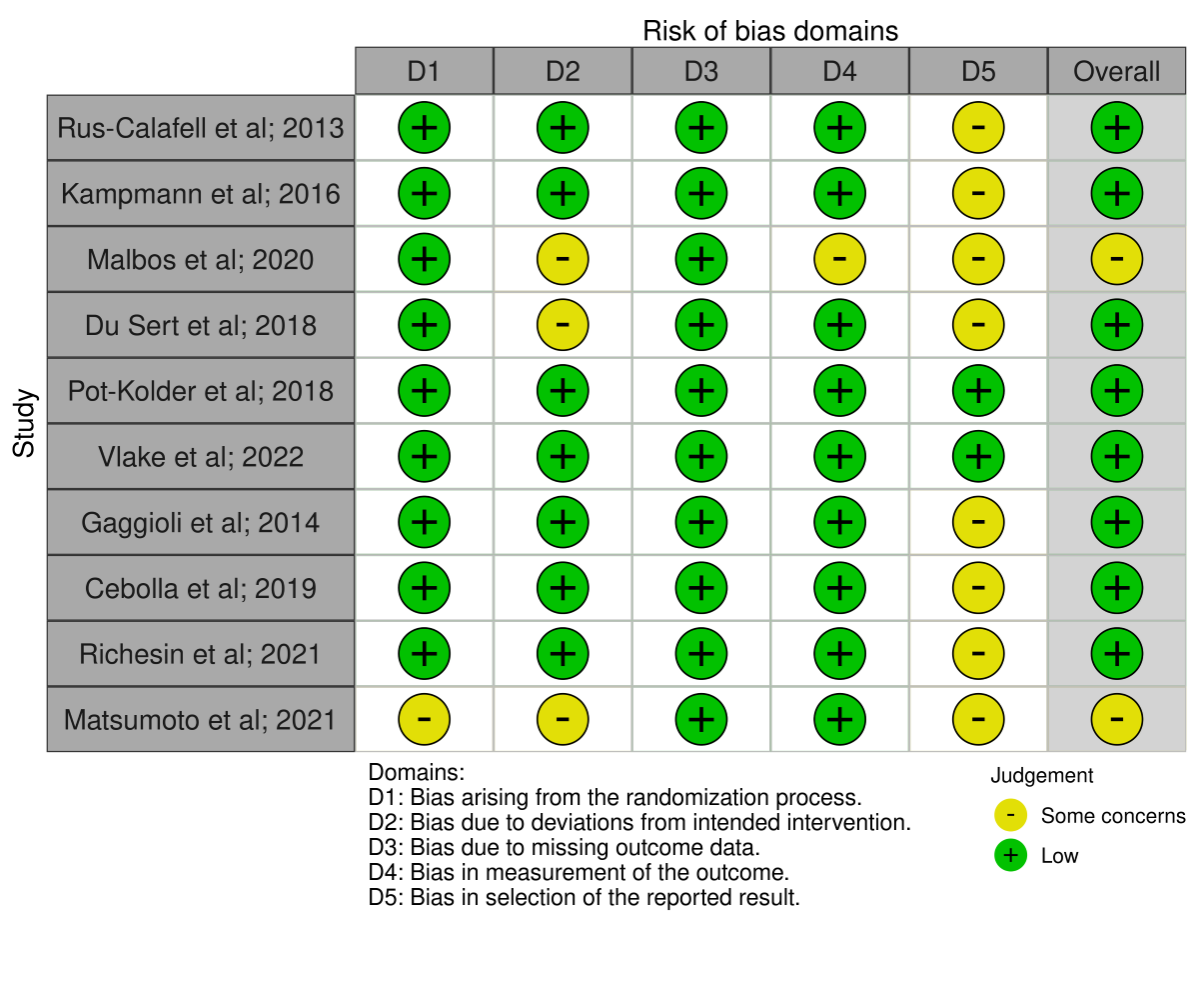
Risk of bias carried out using the Cochrane Risk of Bias Tool [29,30,33,34,37,40,41,43-45].

The risk of bias was found to be low for both between-group and single-arm or within-subject studies. A total of 20% (2/10) [29,30] of the studies assessed using the Cochrane risk-of-bias tool showed some concern relative to the overall risk of bias, and 10% (1/10) of the studies [31] assessed using the Standard Quality Assessment Criteria for Evaluating Primary Research Papers from a Variety of Fields showed some concern related to the quality of the study (see Table 2 and Figure 2 for a summary).

A total of 900 participants received an intervention integrated with the use of VR technologies. The main outcome measures used to evaluate the effects of the VR therapies on symptoms were questionnaires and interviews considered the gold standard in the evaluation of those specific psychopathologies (ie, the Beck Depression Inventory for depression or the State-Trait Anxiety Inventory for anxiety). Some investigations also used general indicators of distress such as the Depression, Anxiety, and Stress Scale or the Patient Health Questionnaire (Multimedia Appendix 1). Conversely, when considering positive functioning, the authors referred to different domains of positivity, including positive emotions, self-compassion, social functioning, and relaxation (see Textbox 1 for a summary). The Positive and Negative Affect Schedule (PANAS) and measures of quality of life were the most commonly used indicators.

**Table 2:** Quality assessment carried out using the Standard Quality Assessment Criteria for Evaluating Primary Research Papers from a Variety of Fields.

Study	Quality assessment
Lindner et al [32], 2020	0.95
Geraets et al [35], 2019	0.86
Jones et al [31], 2020	0.5^a^
Tang et al [36], 2021	0.77
Falconer et al [38], 2016	0.95
Habak et al [39], 2020	0.86
Thompson et al [42], 2020	0.95
Chan et al [46], 2021	1
Desai et al [47], 2021	0.9
Riva et al [48], 2021	1

^a^High risk of bias.

**Summary of scales for positive functioning used in the included studies.**

**Specific phobia**

- Rus-Calafell et al [33]: Life Interference Scale
- Lindner et al [32]: Brunnsviken Brief Quality of Life Scale

**Social anxiety disorder**

- Kampmann et al [34]: EQ-5D
- Geraets et al [35]: Manchester Short Assessment of Quality of Life (MSAQ)

**Generalized anxiety disorder**

- Malbos et al [30]: 12-item Short Form Health Survey

**Posttraumatic stress disorder**

- Jones et al [31]: Difficulties in Emotion Regulation Scale (DERS)
- Tang et al [36]: DERS and qualitative analysis
- Vlake et al [37]: 36-item Short Form Health Survey, EQ-5D, and health-related quality of life

**Depression**

- Falconer et al [38]: Quality of Life Enjoyment and Satisfaction Questionnaire–Short Form (QLS-SF)
- Habak et al [39]: Short Warwick-Edinburgh Mental Well-being Scale

**Psychosis and schizophrenia**

- du Sert et al [40]: QLS-SF
- Pot-Kolder et al [41]: MSAQ
- Thompson et al [42]: EQ-5D

**Stress and adjustment disorder**

- Chan et al [46]: Positive and Negative Affect Schedule (PANAS)
- Desai et al [47]: Pittsburgh Sleep Quality Index and qualitative and thematic analysis
- Riva et al [48]: Social Connectedness Scale and Smith Relaxation States Inventory 3
- Gaggioli et al [43]: Satisfaction with Life Scale
- Cebolla et al [44]: PANAS, State Mindfulness Scale, Self-Other Four Immeasurables Scale, and Mindful Self-Care Scale
- Matsumoto et al [29]: psychological check test
- Richesin et al [45]: PANAS

In the following sections, we briefly illustrate the various studies included in this review, each organized under the clinical conditions for which the VR interventions were applied.

### Specific Phobias and Anxiety Disorders

A total of 25% (5/20) of the studies, with 142 participants, were included in this category, ranging from the treatment of specific phobias to generalized anxiety disorders and social anxiety.

#### Specific Phobias

Regarding the treatment of specific phobias, Lindner et al [32] studied the effectiveness of VRET in the treatment of arachnophobia. A total of 25 participants (19 female) underwent automated VRET composed of 8 sequential levels with increasingly realistic and frightening spiders. The results showed a significant effect of treatment on phobia symptoms (*P*<.001; Cohen *d*=1.26) and a small effect on positive functioning (*P*<.001; Cohen *d*=0.49). The results were maintained at the 6-month follow-up. These results suggest that automated VRET applications could be promising automated treatments for this disorder, with a positive effect also on patients’ well-being.

The efficacy of VRET was further supported by Rus-Calafell et al [33], who compared VRET with a classic mental imagery exposure therapy to treat fear of flying. In this case, 15 patients (13 female and 2 male) were randomly assigned to 1 of 2 groups and received either VRET (mean age 37.14, SD 14.28 years; sex, 6/7, 86% female) or mental imagery therapy (mean age 36.13, SD 12.59 years). VRET comprised 3 different scenarios (room, airport, and plane). Participants completed 6 individual treatment sessions of 60 to 75 minutes over a period of 3 weeks and were asked to buy a plane ticket to use during the 15 days following the end of treatment. The results indicated that both groups improved similarly in posttreatment and follow-up assessments. Analysis of the scores obtained immediately after the real flight and 6 months after treatment revealed that the VR group continued to improve on some of the measures (Fear of Flight Questionnaire), whereas the mental imagery group did not; moreover, participants in the VR group experienced less anxiety during the real flight. Regarding positive functioning, the participants in the VR group and the therapists agreed that the degree of severity and interference in the patient’s daily life had decreased, whereas in the mental imagery group, patients reported a significant improvement just in the severity of symptoms and not in interference. In summary, both mental imagery exposure therapy and VRET were effective in treating fear of flying. However, VRET was shown to perform better in the maintenance of outcomes and perceived interference of fear in the participants’ lives.

#### Social Anxiety Disorder

A total of 10% (2/20) of the studies included in this review assessed the potential benefits of VRET in the treatment of social anxiety disorder (SAD). In total, 50% (1/2) of these studies adopted a 3-arm randomized controlled approach with 3 different groups. Kampmann et al [34] randomly assigned patients diagnosed with an SAD to individual VRET (n=20), individual in vivo exposure therapy (iVET; n=20), or a waiting list (n=20). The treatments proposed by Kampmann et al [34] comprised ten 90-minute sessions twice a week, and the virtual situations provided for the VRET group covered one-to-one and group scenarios designed to provoke anxiety in individuals with SAD. The results of the study showed an improvement from the pre- to postassessment time points for both VRET and iVET but with a greater decrease in symptoms for iVET than for VRET. This trend was also confirmed after the 3-month follow-up. iVET but not VRET improved the positive functioning of the participants as measured using the EUROHIS Quality of Life Scale; however, this difference was not significant after the 3-month follow-up.

The second study [35] assessed the feasibility and potential effect of VR-based cognitive behavioral therapy (VR-CBT) in patients with SAD without a control group. The authors recruited 15 patients with SAD, who underwent up to 16 VR-CBT sessions. Questionnaires regarding clinical and functional outcomes, as well as diary assessments of social activity, social anxiety, and paranoia, were completed at baseline, posttreatment assessment, and the 6-month follow-up. The treatment comprised a 40-minute VR session, and patients were able to explore different virtual scenarios that the therapist could manipulate in terms of crowdedness, ethnicity, intensity, frequency of hostile looks, interpersonal distances, and watching or staring behavior. The VR exercise allowed the patients to test their beliefs and approach or avoidance behaviors while the therapist provided feedback on cognitions and behaviors. The results showed a general improvement in all the outcome measures—social interaction anxiety was significantly reduced at posttreatment compared with baseline assessment (*P*=.008; Cohen *d*=0.9), depression was significantly reduced at posttreatment assessment (*P*=.01; Cohen *d*=1.1), and positive functioning (measured using the Manchester Short Assessment of Quality of Life) increased between baseline and posttreatment assessment (*P*=.02; Cohen *d*=0.5). This improvement was maintained at follow-up.

#### Generalized Anxiety Disorder

Malbos et al [30] assessed the efficacy of VR combined with relaxation in patients with generalized anxiety disorder by comparing VR relaxation therapy with a standard mental imagery exposure therapy. A total of 27 participants (13 female) were randomly assigned to VR relaxation therapy or mental imagery. The therapy was delivered in 6 weekly sessions of 30 minutes—the first 3 sessions were used to teach relaxation techniques that the participants were free to choose and repeat in the remaining 3 sessions. At the beginning of each session, VR relaxation therapy participants could select 6 different relaxing virtual situations to exercise the relaxation techniques. The results showed significant improvements in anxiety, worry mood, and positive functioning in both groups, although the 2 groups were not statistically different.

In summary, VRET has proven to be effective, useful, and user-friendly for patients with phobias and anxiety disorders in reducing the related symptomatology. However, further research is needed to draw inferences about the effectiveness of this advanced technology in improving positive functioning as the data regarding this domain are still weak, with 20% (1/5) of the studies included in this category not showing any improvement or additional advantage in promoting well-being for VRET compared with standard in vivo therapies.

### PTSD Symptoms

A total of 15% (3/20) of the studies examined the effect of a VR program on posttraumatic stress symptoms, as well as indicators of positive functioning. The first 67% (2/3) of these articles described a single research trial, not published yet. The third article (1/3, 33%) described an intervention aimed at preventing the long-term posttraumatic psychological consequences of being hospitalized in the intensive care unit (ICU).

Jones et al [31] presented the initial data of a new computer-assisted rehabilitation virtual environment developed to treat combat-related PTSD symptoms in the first 11 patients recruited for this RCT study. The multimodal motion-assisted memory desensitization and reconsolidation (3MDR) therapy consists of six 90-minute sessions where participants initially walk on a treadmill (placed inside a virtual room) while listening to self-selected music reminiscent of their military deployment. Then, 1 to 7 images are projected, and the participant has to describe the traumatic scenario as well as the associated physical sensations, emotional words, and thoughts. A ball displaying a series of numbers (which the participants read out loud) briefly appears, moving back and forth horizontally across the screen. This cycle is repeated for all 7 images. Jones et al [31] demonstrated that 3MDR significantly improved PTSD symptoms and emotion regulation strategies (assessed using the Difficulties in Emotion Regulation Scale).

A further pilot study with the same VR intervention was published [36], with a specific focus on the issue of emotion regulation. In this study, 9 participants were interviewed, and the qualitative analyses of these interviews were correlated with their scores on the Difficulties in Emotion Regulation Scale. After the intervention, participants reported better emotion regulation, with an increased ability to recognize, accept, and cope with emotions (positive and negative).

The third study, conducted by Vlake et al [37], explored the effects of an ICU-specific VR (ICU-VR) program on the psychological distress and quality of life of 89 patients hospitalized in the ICU because of COVID-19 infection. After hospital discharge, they were assigned to the ICU-VR intervention to prevent the onset of possible posttraumatic symptoms. The intervention consisted of watching a 14-minute–long informational video in VR, where the person was welcomed into the ICU and every machine and procedure was described in detail. The results of this investigation showed that ICU-VR improved patients’ perceived quality of, satisfaction with, and rating of ICU aftercare and decreased psychological distress up to 6 months after hospital discharge. However, the intervention did not significantly improve psychological recovery or quality of life.

### Depression

In total, 10% (2/20) of studies addressing depression were included in this review. They involved 94 patients and applied 2 different VR interventions.

In the first study, Falconer et al [38] addressed self-criticism in a sample of 15 individuals with depression (10 female and 5 male; mean age 32 years) by promoting self-compassion. The authors developed an immersive VR scenario (a virtual room) in which participants could interact compassionately with a crying virtual child while in a virtual adult body. In the second phase of the protocol, participants were in the child’s body and could experience a recording of their compassionate gestures and words being delivered to them from this first-person perspective in the body of the child. The same protocol was repeated for a total of 3 weekly sessions. Depressive symptoms, self-compassion and self-criticism were also assessed (Multimedia Appendix 1). By having participants in an adult and then a child virtual body in succession, the authors documented that the VR scenario effectively provided a self-to-self situation, enabling participants to deliver compassionate sentiments and statements to themselves. After each session, patients increased their recognition of the self in the body of the adult and the child, and they reported feeling comforted while in the body of the child. Statistical analyses revealed a significant linear decrease in depressive symptoms (measured using the Patient Health Questionnaire–9) from baseline to follow-up, with over half of the patients reporting reliable levels of improvement. The self-compassion scale demonstrated improvements at postintervention measurement, whereas self-criticism decreased significantly. Considering its brevity (3 sessions), the authors concluded that this VR scenario could be easily integrated into traditional psychotherapies to treat individuals with depression [38].

In the second study included [39], a new VR program—Edge of the Present—was developed and applied to promote optimism and future thinking in 79 individuals with depression (53 female, 23 male, and 3 intersex), with an average age range of 25 to 34 years. They were assessed with a pretest-posttest research design using the PANAS, Short Warwick-Edinburgh Mental Well-being Scale, and Beck Hopelessness Scale. The VR protocol included a single 10-minute session with the Edge of the Present software. It consisted of a sparsely furnished room with doors and windows that participants explored freely. Edge of the Present is designed to reward such exploration with positively experienced imagery. Hence, the door opens onto a series of spectacular immersive landscapes (7 different vistas, including alpine scenes, lush rainforest, tropical beaches, and a desert) accompanied by environmental effects such as a warm breeze, intensifying the sensory experience. The greater the engagement with the room (ie, opening and closing a door or window), the more increasingly enriched the bare room becomes by the outside landscape (ie, ferns growing inside the room). Edge of the Present provokes a sense of hopeful anticipation—each time the door is opened, there is a new landscape for the user to experience and be incorporated into their world (the room). Thus, through their virtual explorations, the user learns both that openness and curiosity lead to positively reinforcing experiences and that elements of these experiences and environments become integrated into the room they are inhabiting (ie, to enrich the internal world of the user). The primary outcome measure in this study was hopelessness as measured using the Beck Hopelessness Scale, which decreased significantly from pre- to postintervention measurement. Changes in positive and negative mood and increases in well-being were measured using the PANAS and the Short Warwick-Edinburgh Mental Well-being Scale. These results suggest that 10 minutes within the immersive virtual environment can have a positive impact on mood and a significant increase in well-being following the participant’s involvement in Edge of the Present.

These 2 investigations showed that the VR protocol developed to treat depressive symptoms can also have a beneficial effect on different areas of positive functioning, from self-compassion to positive emotions and subjective well-being, in line with the positive mental health approach.

### Psychosis and Schizophrenia

In total, 15% (3/20) of the studies evaluated the benefits of VR treatment in patients with schizophrenia or other psychotic disorders. A total of 150 patients were included, and 3 different VR programs were tested.

du Sert et al [40] used an RCT design to test the effect of a VR intervention using an avatar to treat patients with drug-resistant schizophrenia and compared them with a treatment-as-usual condition. The intervention consisted of 7 weekly sessions; the first one was dedicated to the creation of a specific avatar for each patient that represented their most recurrent persecutor. In sessions 1 to 3, the therapist induced a dialogue between the patients and their avatars to improve emotional regulation and assertiveness. Self-esteem was emphasized in session 4, reinforced by enabling the patients to express themselves and consider their personal qualities. In the final consolidation sessions, patients were encouraged to apply what they had previously learned. Over the course of the therapy, the avatar’s interaction with the patient became gradually less abusive and more supportive. The authors assessed both symptoms and positive functioning before and after the intervention and at follow-up and concluded that the beneficial effect of the VR intervention consisted in changing the way patients relate and respond to their voices by tackling emotional regulation, enhancing self-esteem, and promoting acceptance rather than directly challenging beliefs about the voices [40].

In another RCT, Pot-Kolder et al [41] addressed the issues of paranoid ideation and social functioning in a sample of 116 patients with psychosis; 58 of them were randomized to receive VR-CBT treatment, and 58 were randomized to the waiting list. The VR-CBT consisted of 4 virtual social environments (a street, bus, café, and supermarket) where various avatars were placed to interact with the patients. The therapist could vary the number of human avatars (0-40), the characteristics of the avatars (including sex and ethnicity), and the avatars’ responses to the patient (neutral or hostile and eye contact) to match the paranoid fears of the patient. Patients and therapists communicated during VR sessions to explore and challenge suspicious thoughts during social situations, drop safety behaviors during social situations, and test harm expectancies. No homework exercises were given between sessions. The treatment included 16 sessions. The primary outcome of this RCT was patients’ social participation, which did not change significantly after treatment. The same nonsignificant change was also observed for positive functioning, but social functioning and paranoid ideation significantly improved in the VR-CBT condition compared with the waiting list [41].

Similarly, the third investigation addressed improving social-cognitive functioning in 19 patients in the early stages of psychosis. It was a pilot study in which a virtual world environment platform (*Second Life*) was used to adapt a traditionally face-to-face–delivered social cognition and interactional training. The social cognition and interactional training–VR intervention consisted of 10 sessions (2 individual and 8 group sessions with 3-5 participants). The first 3 sessions focused primarily on emotion recognition, the next 3 sessions focused on attribution bias and paranoia as an emotion, and the last 2 sessions focused on “skills acquisition” using a cognitive behavioral therapy (CBT) framework to discuss examples of social difficulties faced by the participant. After the intervention, a significant increase in emotion recognition and a significant decrease in the anxiety and depression subscale of the EQ-5D were observed. This is the first study to use a virtual world to deliver structured group therapy in early psychosis with a specific focus on social interactions. It documented a positive effect with good feasibility and acceptability from participants [42].

In summary, the 3 VR protocols applied in the treatment of patients with psychosis demonstrated to have a beneficial effect on their paranoid symptoms and also a positive effect on self-esteem, acceptance, and emotion regulation strategy. However, the positive effect on social interactions in the real world has yet to be confirmed.

### Stress and Adjustment Disorder

A total of 35% (7/20) of protocols using VR interventions for addressing stress were included in this review, with a total of 403 participants.

The first protocol is the one used by Gaggioli et al [43] in the context of stress-associated disorders. The authors evaluated the effectiveness of an interreality protocol for the prevention and management of psychological stress compared with stress management training based on CBT and a waitlist control group in a sample of 121 workers (61 high school teachers and 60 nurses) with high levels of perceived stress and low levels of self-efficacy. The interreality protocol included virtual experiences—controlled by the therapist and focused on learning coping skills and improving self-efficacy—and specific real-world experiences where the person’s behaviors and emotions were constantly monitored through the use of wearable biosensors and smartphones to assess the situation and improve the coping skills used in real time. After a 5-week treatment (10 sessions, 2 times per week), reductions in perceived stress and improvements in coping skills were observed in both conditions (interreality and CBT); however, the use of interreality resulted in a significant reduction in chronic “trait” anxiety and a significantly greater increase in emotional support skills compared with CBT (for more details on the outcome measures, see Multimedia Appendix 1).

The second protocol identified is the one used in the study by Cebolla et al [44] in compassion-based interventions. The study involved 16 college students (mean age 30.56, SD 10.86 years) who participated in a self-compassion meditation supported or not (control condition) by an embodied VR system. The Machine To Be Another (TMTBA) is a VR system based on a body swap, and this study involved the use of a visor (VR Oculus Rift) that remotely controlled a camera placed in front of the torso of an actor trained to imitate the performer’s precise movements. The system allows the person to identify with another person’s body, whose torso, legs, and arms they can see. In the TMTBA-VR condition, the self-compassion meditation was played to the participant for 15 minutes, allowing a third-person perspective of oneself. The study showed that following a 2-week meditative practice yielded increased awareness and attention to mental events and bodily sensations, with no differences between the groups. Furthermore, the VR condition yielded an increase in positive affect toward oneself and self-care behaviors, which were significantly higher than those in the traditional meditation condition. Finally, in the TMTBA condition, adherence to meditative practice after 2 weeks was higher in participants with lower visual imagery. According to the authors, these data emphasize the important role that VR could play in psychological interventions where mental imagery is often used (see Multimedia Appendix 1 for more details on the outcomes measured). The results obtained by Cebolla et al [44] showed the positive role of VR in compassion-based interventions, emphasizing the need for future studies on this topic.

VR was also found to be effective in managing stress without the guidance of a counselor, as demonstrated by the Digital–Structured Association Technique (SAT) method developed and evaluated by Matsumoto et al [29]. The Digital-SAT method is an adaptation of a stress management technique used in SAT therapy and consists of re-enacting a stressful physiological response and then counterbalancing it with the visualization of pleasant images using VR technology, which leads to a reduction in discomfort. The intervention protocol involved the combined use of 2 apps to implement the Digital-SAT method: the VR app, aimed at reducing emotional stress by displaying pleasant images through an HMD, and the chat bot app, aimed at improving the continuity of the intervention through an automated chatbot. Matsumoto et al [29] found that the combined use of the VR app and the chat bot app produced a better emotional stress reduction effect after 4 weeks, and they encouraged the continuous implementation of the intervention in a sample of 70 nurses exposed to mental and physical strain and pressure (see Multimedia Appendix 1 for detailed outcome measures). The Digital-SAT method can be considered a promising tool for improving self-care autonomously even in work and daily life settings as it does not necessarily require the involvement of a therapist.

Another area where the application of VR aims to promote positive functioning and manage stress is the context of art. In this regard, Richesin et al [45] compared 2D and 3D art making on measures of stress, anxiety, and mood in a sample of 44 psychology students. Specifically, the 3D intervention involved the use of an HMD (Oculus Quest) and a drawing app (Google Tilt Brush), which participants used to draw freely for a period of 15 minutes using any available tools. This condition was compared with a traditional art-making intervention, and the main outcomes of this study were physiological and self-report measures of stress, anxiety, and well-being (see Multimedia Appendix 1 for more details on the outcome measurements). The study showed that drawing freely using VR can lead to a reduction in negative affect and anxiety levels (state and trait) similar to the effect of the traditional method of art making, but only the 3D group showed a significantly greater decrease in heart rate than the control group. The authors suggested that VR could be a useful tool in art therapy, but the results may be a consequence of the novelty effect.

Another 15% (3/20) of studies examined the effectiveness of VR in stress management during the COVID-19 pandemic. The study by Chan et al [46] involved 2 experiments that explored and compared the effects of VR nature and VR urban environments on the mental condition of individuals who experienced isolation because of COVID-19. The protocol consisted of 7-minute sessions of exposure to both urban and natural environments once per week. A total of 30 students participated in the first study, and 20 older adults participated in the second one. Their positive and negative affect, as well as their connectedness with nature, were assessed before and after treatment. The results from the student and older adult groups showed that walking in a virtual forest decreased stress and negative affect owing to its connection with nature, although no effect was observed on positive affect.

The study designed by Desai et al [47] explored whether using a virtual heart-based meditation program was associated with the improvement of stress levels and quality of sleep during the COVID-19 pandemic. A total of 63 participants underwent 1 weekly virtual trainer-guided group heartfulness relaxation and meditation session for 8 weeks while self-practice was recommended. The results showed a decrease in stress, assessed using the Perceived Stress Scale, and improvements in sleep quality, assessed using the Pittsburgh Sleep Quality Index.

The aim of the last study (Riva et al [48]) was to evaluate the effectiveness of a VR protocol (*the “Secret Garden” 360-degree VR experience*) to assist individuals in coping with the psychological burden related to the COVID-19 pandemic, improving their well-being, and reinforcing social connectedness. In each session, 40 participants who had experienced at least 2 months of quarantine could travel through a virtual garden for 10 minutes for a total of 7 sessions once per week. The results showed that the intervention was associated with improvements in depression and stress but not in perceived hopelessness. There was also a significant increase in social connectedness.

In summary, VR protocols were effective in reducing stress and promoting self-care and positive coping skills also without the presence of a therapist. However, the VR protocols and assessment measures used were very heterogeneous.

## Discussion

### Principal Findings

This review aimed to collect and summarize the literature on the application of positive mental health interventions integrated with VR that yielded beneficial effects for both symptoms and indicators of positive functioning. The first observation derived from this review is the paucity of studies that assessed both distress and well-being despite the increasing number of published articles in recent years (Table 1) and the increasing use of digital technologies in mental health practice. A possible explanation for this phenomenon is that positive outcomes are not widely evaluated in research and clinical practice compared with the assessment of symptoms or indicators of distress. This may represent an existing imbalance between the traditional and the positive psychology perspective [7,9] or simply the need to limit the survey burden and time dedicated to assessment in current research. In any case, we included only 20 investigations over 10 years of research.

Starting with anxiety and phobias, the use of VR in this field mainly involves traditional exposure therapy delivered through VR devices (VRET). Although exposure therapy is a traditional and effective method to treat anxiety disorders, we included studies that applied this technique supported by VR devices as exposure implies behavior changes and opportunities for learning new skills, which are strongly associated with increases in self-efficacy and mastery [51]. Various authors have suggested that exposure therapy may indirectly promote positive characteristics such as courage, persistence, goal setting, and planning and could trigger positive affect, a sense of pride, and satisfaction once the exposure activity is successfully performed [6,24,52]. Thus, it can be included under the wide umbrella of positive interventions [7,9]. Exposure therapy is generally delivered in the presence of a clinician, but 20% (1/5) of the included studies [32] used an automated exposure protocol. All the included protocols (5/5, 100%) were effective in reducing the symptomatology of the patients, but they were not equally effective in improving their positive functioning. The results of the study by Rus-Calafell et al [33] showed an improvement in the interference of the phobic object in life only for the VR therapy compared with the imagery control condition, whereas Kampmann et al [34] found that VR therapy did not improve positive functioning at all as compared with a waitlist control. Finally, Malbos et al [30] showed that VR relaxation improved positive functioning but not more than a classic imagery intervention. From this picture, VRET seems to be effective and useful for patients in reducing the symptomatology related to phobias and anxiety disorders (see also the studies by Baghaei et al [1], Schroeder et al [2], and van Loenen et al [3]). However, further research is needed to draw inferences about the effectiveness of this advanced technology in improving positive functioning as the data regarding this domain are still inconclusive.

The same inferences can also be drawn for other mental health problems analyzed in this review. For instance, for PTSD, we only found 15% (3/20) of studies that assessed positive functioning (ie, emotional regulation) together with symptomatology. In total, 67% (2/3) of these studies referred to the same intervention protocol, consisting of exploring a virtual room on a treadmill followed by the presentation of 7 images associated with 3MDR, which can be considered a technologically advanced form of traditional trauma intervention—exposure therapy and eye movement desensitization and reconsolidation [53]. The results of this VR intervention were promising—PTSD symptoms improved, together with better use of emotion regulation strategies. Participants reported a better ability to recognize, accept, and cope with emotions after treatment. However, these studies involved only a few patients who were treated with one specific VR protocol. Similarly, the results of the study by Vlake et al [37] showed a decrease in distress among COVID-19 ICU survivors but not a significant improvement in the positive functioning of the patients.

The studies on depression were based on different types of protocols, but also in this case, the findings regarding improvements in positive functioning are inconclusive. One of the studies focused on self-compassion, and participants were required to compassionately interact with an avatar first and then re-experience the effect of their interaction by taking on the role of the child avatar. In the second study, participants had to explore a virtual room, and such exploration was rewarded with positive experiences as the virtual doors opened onto a series of spectacular immersive landscapes. Both studies showed promising results and highlighted the benefits of the protocols in decreasing the severity of depressive symptoms. However, concerning positive functioning, the results of the first treatment showed that the protocol did not yield any change in self-compassion, only in self-criticism, whereas the second intervention yielded an improvement in individuals’ positive affect and a significant increase in well-being following the participants’ involvement in the virtual procedure. The first investigation contributes important data on the crucial differentiation between symptom improvements (ie, a decrease in self-criticism) and the promotion of positive functioning (ie, an increase in self-compassion). These findings emphasize the need to implement interventions specifically focused on well-being promotion and not merely on symptom reduction [6,7,54,55].

The second VR intervention, in contrast, shares the same theoretical framework of traditional behavior activation therapy, where positive and rewarding activities are prescribed to help patients with depression experience pleasure and engagement in their lives [56,57]. Hence, both investigations confirm the observation that depressive disorders can be addressed by changing the complex balance between positive and negative affect [58,59]. VR may play an important role in this regard, but further studies are needed to develop the most suitable protocols for enhancing well-being in individuals with depression.

VR treatments for individuals with psychosis and schizophrenia were focused on social functioning and positive relationships. A total of 15% (3/20) of the studies included in this review were based on social interaction with one or more avatars to improve social interactions and cognitive functioning. du Sert et al [40] showed that the VR intervention was effective in changing the way patients related and responded to their voices by tackling emotional regulation, enhancing self-esteem, and promoting acceptance as compared with treatment as usual. These results are in line with those of Thompson et al [42], who found a significant increase in emotion recognition and a significant decrease in anxiety and depression in their pilot study. In contrast with these results, Pot-Kolder et al [41] did not register a significant change in patients’ social participation and positive social functioning compared with the waitlist condition. Only the dimensions of social functioning and paranoid ideation improved when compared with the control group. However, it should be noted that the authors used the same VR intervention that Geraets et al [35] used in the treatment of individuals with SAD, and in this case, the intervention was able to show an improvement in social functioning. When the protocol was delivered to patients with psychosis, findings showed that the intervention based on a one-to-one or group interaction with an avatar yielded general beneficial effects on paranoia and other psychotic symptoms but not necessarily on the social participation and positive interpersonal functioning of patients (see the study by Schroeder et al [2] for a review). Further research is needed to clarify whether the beneficial effect of the VR intervention might be linked to the severity of the clinical condition of participants, their cognitive bias, their preexisting social skills, or other peculiar issues yet to be investigated [60,61].

Outside the domain of psychiatric conditions, this systematic review analyzed VR interventions aimed at promoting well-being and reducing stress, including COVID-19 pandemic distress. As many protocols involving the use of VR are being developed in this field, a summary of their findings might be useful to understand whether these interventions were beneficial in decreasing the stress and anxiety related to the virus and social isolation and in restoring well-being after the pandemic. In total, 10% (2/20) of the included studies [46,48] were based on a virtual natural scenario that the participant could explore, whereas another protocol reproduced an ICU in which patients were hospitalized because of COVID-19. The interventions with virtual natural scenarios were shown to be effective in reducing stress, negative affect, and depression and increasing social connectedness, in line with the well-established association between feelings of well-being and being in contact with nature (in this case, through a virtual landscape) [62-64]. However, the protocol by Chan et al [46] did not yield an improvement in positive affect, and the one by Riva et al [48] was not effective in improving perceived hopelessness. Finally, Desai et al [47] proposed a heart-based relaxation protocol that showed a decrease in stress and a better quality of sleep. These data support the use of VR in addressing the stress and negative feelings associated with the pandemic, but its beneficial effects for restoring well-being have not been confirmed, with the sole exceptions of social connectedness and sleep quality.

Other than stress related to COVID-19 and the pandemic, VR interventions also showed their utility in relieving and preventing stress more generally. Gaggioli et al [43] showed that a VR exposure-based therapy prompted a significant reduction in perceived stress and improvements in coping skills in workers. Moreover, the group that underwent the VR intervention showed a reduction in chronic “trait” anxiety and a significantly greater increase in emotional support skills. Compassion-based interventions have also been developed and delivered using VR, and the study by Cebolla et al [44] showed that this intervention was effective in increasing positive attitudes toward the self and others and decreasing negative qualities toward the self. However, the data do not fully support an additional benefit of using VR compared with traditional meditation techniques. Finally, 10% (2/20) of the studies included in this review showed that VR interventions were effective in reducing emotional stress also without the guidance of a counselor [29] and in reducing negative affect and anxiety levels (state and trait) using virtual art therapy [45]. In summary, when considering nonclinical populations such as workers or college students, VR interventions showed their utility and effectiveness in reducing stress and promoting positive function (ie, emotional support skills and compassion toward others and the self) even without the guidance of a therapist or counselor. These data are very promising considering the large scalability of these VR protocols after the pandemic and among the general population [65]. However, solid evidence that VR interventions are more effective compared with traditional ones (ie, mindfulness, traditional CBT, relaxation, and art making) is still to be completely demonstrated.

### Limitations

One of the main limitations of this review was its heterogeneous nature. It included different populations (clinical and nonclinical) treated with many different VR protocols and evaluated using different outcome measures. This is particularly true for the assessment of positive functioning, where no consensus emerged among investigations with the sole exceptions of the PANAS and the EQ-5 used EUROHIS Quality of Life Scale in 15% (3/20) of the studies. Hence, there were not enough quantitative data to support a meta-analysis. Accordingly, this work aims to provide a summary of the progress made in the last decade and delineate future directions to follow for researchers and clinicians interested in promoting positive mental health through VR interventions.

Another limitation of this study was the absence or exclusions of specific clinical domains (such as OCD, eating disorders, and addiction-related disorders), where many other VR protocols have been recently developed [11,12]. For OCD, we found no studies that assessed positive functioning along with negative symptoms. We decided not to include eating disorders and addiction-related disorders as the positive functioning of these clinical populations was found to be intertwined with their disorders [23,25,26]. Thus, when analyzing the effects of VR interventions, these clinical conditions might need to be considered from a different perspective.

Finally, the studies included in this review account only for adult populations, leaving out children and older individuals. The reason for this choice lies in the fact that these populations present specific features in terms of mental health; in older adults, core issues are correlated with memory loss and physical and cognitive decline, whereas in children and adolescents, issues such as self-esteem, identity, and personality development are the main challenges associated with distress and well-being. Moreover, the features of psychological distress and well-being in these populations are strongly influenced by age and the stage of development or aging [66,67]. We found that many studies that used VR to promote the mental health of children and older adults [18-21] focused on aspects that differed from those in studies on adults, such as neurological and cognitive development or decline, respectively.

### Conclusions and Future Directions

In conclusion, this review provides robust evidence supporting the beneficial effect of VR therapy in improving stress and negative symptoms. However, in participants, the impact of VR treatments on positive functioning remains unclear, with 35% (7/20) of the studies showing no or a small effect on various dimensions of positivity, ranging from positive affect to relaxation, self-compassion, and social interaction. The variety of outcome measures included and their different sensitivities to clinical changes might be the reason why the results on the beneficial effect of VR interventions in promoting positive functioning are still inconclusive [68]. It is of crucial importance that future research systematically addresses the impact of VR interventions also on individuals’ positive functioning to identify the most suitable protocols for enhancing well-being in individuals with different mental health conditions (from those with psychiatric disorders to those dealing with stress or the negative consequences of the recent COVID-19 pandemic). Indeed, our review showed that VR interventions applied in the general population were effective both in addressing stress and promoting positive mood, social connectedness, and better sleep quality. These data suggest that VR interventions might be cost-effective and largely scalable, particularly when they do not require the guidance of a counselor or therapist.

However, this review also suggested that the beneficial effect of VR interventions might be linked to the severity of the clinical condition of participants (ie, the same VR protocol with the interaction with an avatar was able to improve social functioning in individuals with social anxiety but not in patients with paranoia). Previous work has documented the peculiar combinations of personal resources and vulnerabilities that characterize clinical populations [23,25,66]. In particular, it was found that clinical populations generally present impairments in well‐being, and positive interventions developed within the positive psychology perspective were found to be effective in addressing those impairments [7,28]. Owing to its flexibility and capacity to engage participants, VR interventions might play a crucial role in promoting better mental health in the population, including clinical samples. Considering the large number of private and public investments in digital technologies for mental health, this review suggests further research to develop existing VR software and treatments to align them with the modern positive mental health approach.

## Appendix

Multimedia Appendix 1 Summary of the studies included in the review.

## Abbreviations

3MDR: multimodal motion-assisted memory desensitization and reconsolidation
CBT: cognitive behavioral therapy
HMD: head-mounted display
ICU: intensive care unit
ICU-VR: intensive care unit–specific virtual reality
iVET: individual in vivo exposure therapy
OCD: obsessive-compulsive disorder
PANAS: Positive and Negative Affect Schedule
PRISMA: Preferred Reporting Items for Systematic Reviews and Meta-Analyses
PTSD: posttraumatic stress disorder
RCT: randomized controlled trial
SAD: social anxiety disorder
SAT: Structured Association Technique
TMTBA: The Machine To Be Another
VR: virtual reality
VR-CBT: virtual reality–based cognitive behavioral therapy
VRET: virtual reality exposure therapy

## Data Availability

Data sharing does not apply to this paper as no new data were created or analyzed in this study.
