# Supplementary material for: The Use of Virtual Reality Interventions to Promote Positive Mental Health: Systematic Literature Review
Source: JMIR Ment Health. 2023 Jul 6;10:e44998. doi: 10.2196/44998 (PMC10360019; doi:10.2196/44998)
Supplement: Multimedia Appendix 1 [file mental_v10i1e44998_app1.docx]

Table 1: Summary of the studies included in the review. Included Questionnaires Abbreviations: Anxiety Disorders Interview Schedule (ADIS-V), Subjective Units of Discomfort (SUD), Beck Depression Inventory–II (BDI-II), The Questionnaire Upon Mental Imagery (QMI,) Presence and Reality Judgment Questionnaire (PRJQ), Fear of Flying Questionnaire (FFQ), Fear of Flying Scale (FFS), Danger Expectations and Flying Anxiety Scales (DEFAS), Life Interference Scale (LIS), Patient Health Questionnaire (PHQ-9), Generalized Anxiety Disorder (GAD-7), Fear of Spiders Questionnaire (FSQ), Brunnsviken Brief Quality of life scale (BBQ), Gatineau Presence Scale (GPS), Liebowitz Social Anxiety Scale-Self Report (LSAS-SR), Fear of Negative Evaluation Scale-Brief Form (FNE-B), Depression Anxiety Stress Scale (DASS), Personality Disorder Belief Questionnaire (PDBQ), Eurohis Quality of Life Scale (EQ-5D), Social Interaction Anxiety Scale (SIAS), Manchester Short Assessment of Quality of Life (MSAQ), Experience sampling method (ESM), State Trait Anxiety Inventory (STAI), Penn State Worry Questionnaire (PSWQ), SF-12 Quality of life questionnaire (SF-12), Presence Questionnaire (PQ), Simulation Sickness Questionnaire (SSQ), Clinician Administered PTSD Scale (CAPS-5), PTSD Checklist (PCL-5), Difficulties in Emotion Regulation scale (DERS-18), Zung Self-Rating Depression Scale (SDS), Self-Compassion and Self-Criticism Scale (SCCS), Fears of Compassion Scales (FCS), Virtual Reality Experience Questionnaire (VREQ), Short Warwick–Edinburgh Mental Well-Being Scale (SWEMWBS), Beck Hopelessness Scale (BHS), The Positive and Negative Affect Schedule (PANAS) Psychotic Symptoms Rating Scale (PSYRAT); Beliefs About Voices Questionnaire-Revised BAVQ-R; Positive And Negative Syndrome Scale (PANSS); Quality of Life Enjoyment and Satisfaction Questionnaire-Short Form (QLS-SF), Safety Behaviour Questionnaire-Persecutory Delusions (SBQPD), Paranoid Thoughts Scale (PTS), Brief Psychiatric Rating Scale (BPRS), Social Cognition Screening Questionnaire (SCSQ), Cognitive Style Questionnaire-Short Form (CSQ-SF), Personal and Social Performance Scale (PSP), Bell Lysaker Emotion Recognition Task (BLERT), Social Cognition Psychometric Evaluation (SCOPE), National Adult Reading Test (NART), igroup presence questionnaire (IPQ), connectedness to nature scale (CNS), Perceived Stress Scale (PSS), Pittsburgh Sleep Quality Index (PSQI), Social Connectedness Scale (SCS), Fear of Coronavirus (FCOR), Smith Relaxation State Inventory 3 (SRSI3), Subjective Units of Distress Scale (SUDS), SF-36 Quality of life questionnaire (SF-36), health-related quality of life (HRQoL), Impact of Event Scale-Revised (IES-R; PTSD), Hospital Anxiety and Depression Scale (HADS), Psychological Stress Measure (PSM), Satisfaction With Life Scale (SWLS), Coping Orientation to the Problems Experienced—New Italian Version (COPE-NIV), Betts’ Questionnaire Upon Mental Imagery (QMI), State Mindfulness Scale (SMS), Self-Other Four Immeasurable Scale (SOFIS), Mindfulness Self-Care Scale (MSCS), Adherence questionnaire, Embodiment in TMTBA Questionnaire (TMTBA).

| Study | Sample | Age | Self-Report measures | Treatment Conditions | Follow Up | Main Findings | Limitation |
| --- | --- | --- | --- | --- | --- | --- | --- |
| **Specific Phobia** |  |  |  |  |  |  |  |
| Rus-Calafell et al. (2013)  Pilot Study | 15(13 women) | Mean=36.6 | ADIS-IV, SUD, BDI, QMI, PRJQ, FFQ, FFS, DEFAS, LIS | VRET (n=7)  Vs.  MI (n=8) | 6 months | Improvement in both groups but the VRET showed to perform better in the maintenance of outcomes and the perceived interference of fear in the participant’s life. | small sample size, predominance of women in the sample |
| Linder et al. (2020)  Single arm study | 25(19 women) | Mean=20 | PHQ-9, GAD, FSQ, BBQ, SSQ, GPS | VRET | 6 months | Significant effect on SFQ and BBQ | small sample size, no control group, only self-reported outcome measures, no long-term follow-up. |
| **SAD** |  |  |  |  |  |  |  |
| Kampmann et al. (2016)  Randomized Controlled Trial | 60 | Mean=36.9 | LSAS-SR, FNE-B, DASS, PDBQ, EQ-5D | VRET=20  iVET=20  WL=20 | 3 months | Improvement from pre to post assessment for both VRET and iVET but with a greater decrease for iVET than for VRET. iVET, but not VRET, improved the quality of life of the participants, however this difference was not significant after the 3 months follow up. | social situations in exposure exercises were not identical in both treatment conditions. |
| Geraets et al. (2019)  Pilot Study | 15(8 women) | Mean=34.9 | SIAS, BDI, MSAQ, ESM | VRT | none | Improvements in social anxiety and quality of life were found at post-treatment. At follow-up, the effect on social anxiety was maintained. No increase was observed in social activity. | Lack of control |
| **GAD** |  |  |  |  |  |  |  |
| Malbos et al. (2020)  Randomized Controlled Trial | 27(13 women) | Mean=48.4 | STAI, BDI, PSWQ, SF-12, SUD, PQ, SSQ | VR  MI | none | Significant improvement in anxiety, worry, mood and mental quality of life in both groups. No difference between the two groups | lack of control, high rate of dropout |
| **PTSD** |  |  |  |  |  |  |  |
| Jones at al. (2020)  Single arm study | 11 (10 male) | 30-60 (mean age not provided) | CAPS-5, PCL-5, DERS-18 | VR-3mdr |  | Clinically significant reduction in PTSD symptoms. Improvements in emotion regulation after intervention | Preliminary data, part of a larger RCT not published yet |
| Tang et al. (2021)  Single arm study | 9 | Not reported | DERS -18  And qualitative assessment of quality of life | VR-3mdr | 6 months | Improvements in DERS-18 from pre to post intervention and follow-up. Qualitative analyses correlated these improvements to gaining awareness of emotions, having the correct and appropriate vocabulary to identify emotions, and strategies (including acceptance) to cope with emotions. | Preliminary data, part of a larger RTC not published yet |
| Vlake et al. (2022) Randomized Controlled Trial | 89 (26 women) | Mean= 58 | SF-36, EQ-5D, HRQoL, IES-R, HADS | ICU-VR  Vs.  Control group | 3-months | Results showed ICU-VR was effective in improving patients’ perceived quality of, satisfaction with, and rating of ICU aftercare, however, ICU-VR did not improve psychological recovery or quality of life. |  |
| **Depression** |  |  |  |  |  |  |  |
| Caroline J. Falconer et al. (2016)  Case series | 15 (10 women) | Mean= 32 | PHQ-9, SDS, SCCS, FCS, VREQ | delivering compassion in one virtual body and then experienced receiving it from themselves in another virtual body. | 4-week | Significant reductions in depression severity and self-criticism, as well as to a significant increase in self-compassion, from baseline to 4-week follow-up. Four patients showed clinically significant improvement. | small sample size, predominance of women in the sample |
| Habak et al. (2020) Pilot study | 79 (53women, 3nb) | 18+ | SWEMWBS, BHS, PANAS, Sense of Presence | EOTP | - | Positive mood and well-being increased significantly post-intervention. Hopelessness scores and negative mood decreased, whilst sense of presence was very high. |  |
| **Psychosis and Schizophrenia** |  |  |  |  |  |  |  |
| Du Sert et al. (2018)  Randomized Controlled Trial | 15 (5 women) | Mean= 42.9 | PSYRAT; BAVQ-; PANSS; BDI  QLS-SF | VRT  vs  TAU | 3 months | VRT produced significant improvements in auditory hallucination, depressive symptoms and positive functioning that lasted at the 3-month follow-up period. The therapeutic effects of VRT on the distress associated with the voices were particularly.  strong (d = 1.2). | No active control, few participants, non-blindness of the therapist |
| Pot-Kolder et al. (2018)  Randomized Controlled  Trial | 116 (34 women) | Mean= 38 | SBQPD, PTS, SIAS, BDI, MSAQ | VR-CBT (n=58)  vs  Waiting list (n=58) | 6 months | Paranoid ideation and anxiety were significantly reduced in the VR-CBT group compared with the control group at the post-treatment assessment, and these improvements were maintained at the follow-up assessment. Positive functioning at the post-treatment or follow-up assessments did not differ significantly between groups | No active control, no data on dose-effect, social environment not spontaneous |
| Thompson et al. (2020)  Pilot study | 19 (5 women) | Mean= 26.5 | BPRS, SCSQ, CSQ-SF, EQ-5D, PSP, BLERT, SCOPE, NART | SCIT-VR | none | No significant changes in BPRS; improvements in EuroQual5 (anxiety and depression subscale) | Drop-outs, engagement of patients and some technical difficulties with the VR platform.  No control group |
| **Stress and adjustment disorder** |  |  |  |  |  |  |  |
| Gaggioli et al. (2014)  Randomized Controlled Trial | 121 (73 women) | Mean= 42.5 | PSM, PSS, STAI-Y2, SWLS, COPE-NIV | IR (n=40) Vs. CBT (n=42) Vs. WL (n=39) | none | Significative reduction in perceived stress in both treatments (CBT and IR), but with a significantly greater reduction (12% vs 0.5%) in chronic “trait” anxiety in IR compared with CBT. Both treatments were able to significantly increase most coping skills, but IR group reported a significantly greater increase (14% vs 0.3%) in the Emotional Support skill than CBT. | No follow-up assessment, no measure of physiological stress, no specific evaluation of the effectiveness of the different technological tools included in IR, technology is expensive |
| Cebolla et al. (2019)  Randomized Controlled Trial | 16 (12 women) | Mean= 30,56 | Sociodemographic, Psychological, and Practice-Related Meditation Variables Questionnaire, PHQ-9, GAD-7, QMI, PANAS, SMS, SOFI, MSCS, Adherence questionnaire, TMTBA | TMTBA-VR (n=8) Vs. CAU (n=8) | none | Increased of the positive affect toward self and others and of the frequency of self-care behaviors and decreased of the negative qualities toward self, increased awareness and attention to the present for mental events and bodily sensations after the compassion practice. in both conditions | The sample is small, non-clinical and with high level of education |
| Matsumoto et al. (2021)  Between group design | 70 | Not mentioned | Psychological check test, blood pressure | VR app (n=24) Vs. VR app+CB app (n=23) Vs. CB app (n=23) | none | Significant increase in the self-rating depression and the health counseling needs in the VR+CB group. The group VR app + CB app were more effective in implementing the Digital-SAT method than using the apps separately. | Only 27 of the initial 70 participants remained enrolled until the second blood pressure measurement due their large burden |
| Chan et al. (2021)  Within-subject design | Student 30(21 women)  Elder 20 (18 women) | Stu 20.5 mean  Elder 72.7 mean | IPQ, PANAS, CNS | VR nature exposure vs. Urban nature exposure | - | Results from the young adult sample showed that walking in a virtual forest reduced negative affect due to enhanced nature connectedness and stress | predominance of women in the sample, no follow-up |
| Desai et al. (2021)  Single arm study | 63 (31 women over 36 completed the study) | 18+ | PSS, PSQI | virtual trainer-guided group Heartfulness relaxation and meditation sessions +SELF-PRACTICE | - | There was a significant decrease in PSS and in PSQI between week zero and week eight, regardless of Health Care Professional status. The qualitative thematic analysis strongly supported the survey results. A significant reduction in perceived stress score and improvement in sleep quality index was noted at the end of a virtual Heartfulness meditation program. | No follow-up |
| Riva et al. (2021)  Single arm study | 40 (25 women) | - | DASS-21, PSS, BHS, SCS, FCOR, STAI, SRSI3, SUDS | COVID Feel Good | 2-week | Results showed an improvement in depression, stress, general distress, and perceived stress levels but not for the perceived hopelessness. Moreover, there was social connectedness from T0 to T1 but not a significant reduction in the perceived fear of coronavirus. | No control, in vivo vs. virtual |
| Richesin et al. (2021)  Randomized Controlled Trial | 44 (36 women) | Mean=21.20 | PANAS, STAI, PSS | VR control (n=15) Vs. 2D (n=12) Vs. 3D (n=15) | none | Significant pre- to post-intervention decreases in negative affect and anxiety (state & trait) measures for all groups. Significantly greater decrease in the 3D group in heart rate than control. | Small sample size due to the COVID-19 shutdown, non-clinical and too homogeneous sample, residual change was not incorporated during data analysis, anxiety should have been measured, the pre to post-test design was not ideal for measuring HR and SC, the mood scale employed might have been more specific for mood changes |
